# Four-Year Treatment Outcomes of Adult Patients Enrolled in Mozambique's Rapidly Expanding Antiretroviral Therapy Program

**DOI:** 10.1371/journal.pone.0018453

**Published:** 2011-04-04

**Authors:** Andrew F. Auld, Francisco Mbofana, Ray W. Shiraishi, Mauro Sanchez, Charity Alfredo, Lisa J. Nelson, Tedd Ellerbrock

**Affiliations:** 1 Division of Global AIDS, Centers for Disease Control and Prevention (CDC), Atlanta, Georgia, United States of America; 2 Ministry of Health, National Institute of Health, Maputo, Mozambique; 3 Division of Global AIDS, Centers for Disease Control and Prevention (CDC), Maputo, Mozambique; University of Toronto, Canada

## Abstract

**Background:**

In Mozambique during 2004–2007 numbers of adult patients (≥15 years old) enrolled on antiretroviral therapy (ART) increased about 16-fold, from <5,000 to 79,500. All ART patients were eligible for co-trimoxazole. ART program outcomes, and determinants of outcomes, have not yet been reported.

**Methodology/Principal Findings:**

In a retrospective cohort study, we investigated rates of mortality, attrition (death, loss to follow-up, or treatment cessation), immunologic treatment failure, and regimen-switch, as well as determinants of selected outcomes, among a nationally representative sample of 2,596 adults initiating ART during 2004–2007. At ART initiation, median age of patients was 34 and 62% were female. Malnutrition and advanced disease were common; 18% of patients weighed <45 kilograms, and 15% were WHO stage IV. Median baseline CD4^+^ T-cell count was 153/µL and was lower for males than females (139/µL vs. 159/µL, p<0.01). Stavudine, lamivudine, and nevirapine or efavirenz were prescribed to 88% of patients; only 31% were prescribed co-trimoxazole. Mortality and attrition rates were 3.4 deaths and 19.8 attritions per 100 patient-years overall, and 12.9 deaths and 57.2 attritions per 100 patient-years in the first 90 days. Predictors of attrition included male sex [adjusted hazard ratio (AHR) 1.5; 95% confidence interval (CI), 1.3–1.8], weight <45 kg (AHR 2.1; 95% CI, 1.6–2.9, reference group >60 kg), WHO stage IV (AHR 1.7; 95% CI, 1.3–2.4, reference group WHO stage I/II), lack of co-trimoxazole prescription (AHR 1.4; 95% CI, 1.0–1.8), and later calendar year of ART initiation (AHR 1.5; 95% CI, 1.2–1.8). Rates of immunologic treatment failure and regimen-switch were 14.0 and 0.6 events per 100-patient years, respectively.

**Conclusions:**

ART initiation at earlier disease stages and scale-up of co-trimoxazole among ART patients could improve outcomes. Research to determine reasons for low regimen-switch rates and increasing rates of attrition during program expansion is needed.

## Introduction

Globally, during 2004–2009, the number of HIV-infected patients enrolled on antiretroviral therapy (ART) increased more than 10-fold, from less than 400,000 to more than five million, with most new ART patients enrolled in sub-Saharan Africa [1]. Reporting treatment outcomes of patients enrolled in ART programs is important to demonstrate program effectiveness and justify continued funding, while assessment of factors associated with outcomes can help to identify opportunities for program improvement [2], [3]. Although many sub-national ART programs in sub-Saharan Africa have reported their treatment experience [4]–[12], only a few studies can be considered nationally representative due to the large cohort size [6], [12], or sampling design [13].

In Mozambique, where about 1.6 million people are HIV-infected and about 473,000 need ART [14], numbers of adult patients (≥15 years old) enrolled on ART increased about 16-fold from less than 5,000 to 79,500 during 2004–2007 [15]. We conducted a retrospective, cohort study among a nationally representative sample of adult ART patients starting ART during 2004–2007, to describe mortality rates, attrition rates (numbers of patients who died, were lost to follow-up [LTFU], or stopped ART per 100 person-years), immunologic treatment failure rates, and determinants of attrition and treatment failure. Secondary treatment outcomes of interest were CD4^+^ T-cell (CD4) count response and weight gain. Regimen-switch rates were measured to allow comparison with rates reported from other programs.

## Methods

### Ethics Approval

This study was approved by the Institutional Review Board (IRB) of the United States Centers for Disease Control and Prevention (CDC) and the Mozambican Ethics Review Committee (*Ministerio da Saude Comite Nacional de Bioetica para a Saude*). Both review boards approved the consent procedures. Patient informed consent was not required as only routine, anonymous, operational monitoring data were collected and analyzed.

### Eligibility for ART during 2004–2007

During 2004–2007, patients diagnosed with World Health Organization (WHO) stage IV disease, stage III disease with CD4 counts <350/µL, or stage I or II disease with CD4 counts <200/µL, were eligible for ART [16]. Prescription of trimethoprim-sulfamethoxazole combination, or co-trimoxazole (CTX), was indicated for all ART patients. First-line ART regimens included two nucleoside reverse-transcriptase inhibitors (NRTI) and a non-nucleoside reverse transcriptase inhibitor (NNRTI). Second-line regimens contained a protease inhibitor and two new NRTIs. Before initiating ART, HIV counseling and testing (HIV-CT) of patient partners was recommended to facilitate HIV care for infected partners or prevention interventions for sero-discordant couples.

### Patient Monitoring

At ART initiation, standardized medical records, recommended by the Ministry of Health (MOH), were completed. These records captured, among other variables: date of visit, sex, age at enrollment, date of birth, marital status, employment status, HIV status of partner, WHO stage, CD4 count, weight, height, hemoglobin level, ART regimen and CTX prescription status. At all subsequent visits, the date of the visit, patient weight, clinical stage and ART regimen prescribed were recorded, while at 6-monthly intervals after ART initiation, hemoglobin measurements, and CD4 counts were recommended to monitor disease progression or improvement. Patients collected medications monthly from clinic pharmacies where the date of scheduled antiretroviral (ARV) pick-up appointments and actual ARV pick-up occurrences were documented.

As in other resource-constrained settings where viral load monitoring is not routine [6], [13], [17], during 2004–2008, treatment failure was detected using clinical and immunologic criteria. A new or recurrent stage III or IV condition was considered clinical evidence of possible treatment failure; however, confirmation of failure, with a CD4 measurement, was required before switching regimens. All facilities providing ART had access to CD4 assays. Immunologic failure criteria included a CD4 count decline from baseline, a CD4 count <100/µL, or a 50% decline from peak CD4 count, after ≥6 months of therapy [16]. At the limited number of sites with access to viral load testing facilities, viral load measurements were also encouraged.

### Treatment Outcome Measures

Our reported mortality rate represents the number of documented deaths during ART per 100 person-years of observed therapy. The attrition rate represents the number of patients lost through attrition (death, LTFU, or stopping ART) per 100 person-years of observed therapy. A patient absent from a health facility in the 90 days preceding data abstraction was considered LTFU, unless the medical record stated that the patient had died, stopped ART, or transferred. The date of LTFU was recorded as the date of the most recent visit, or one day after ART initiation if patients only attended the initiation visit. One-year attrition proportions are reported to allow comparison with published literature [11], [18]. Patients transferring to other facilities during the first treatment year were excluded from the one-year attrition proportion. Transfers were censored from time-to-event analyses at the date of transfer.

To allow comparison with other resource-constrained programs [6], [17], we report incidence of the first CD4 count meeting immunologic treatment failure criteria. Incidence of clinical failure is not reported because MOH guidelines required that the often inaccurate diagnosis of clinical failure [19] be confirmed with objective CD4 count measurements before a regimen change was considered. To allow comparison with other programs in resource-limited settings, we also report rates of regimen-switch per 100 patient-years [20].

CD4 counts were determined using FlowCount PLG CD4 assay and analyzed with Beckman Coulter Epics XL-MCL Flow Cytometers (both Beckman Coulter, Inc., Johannesburg, South Africa). Blood Hemoglobins were determined with an automated hematology analyzer, Sysmex XT2000i (Sysmex Europe, Inc., Hamburg, Germany) or HemoCue photometer assay (HemoCue AB, Helsingborg, Sweden).

Similar to other cohort studies in resource-constrained settings [6], adherence to ART was estimated by measuring timeliness of patient visits to scheduled medicine pick-up appointments at clinic-based pharmacies [21]–[23] during the first six months of ART.

### Study Design and Population

A retrospective cohort study design was used. Patient-level data were abstracted from standardized, MOH-recommended medical records onto study questionnaires by trained abstractors in November 2008. Only medical records of adult patients, ≥15 years old at ART initiation, who started ART during 2004–2007, were eligible.

### Sample Size

Sample size calculations were performed using Epi Info™ software (CDC, Epi Info 2008, Version 3.5.1, Atlanta, GA). To achieve a 95% confidence interval (CI) of ±3.0% around the estimate for 6-month attrition, assuming a design effect of 1.5, and a conservative (i.e. higher than expected) 6-month attrition proportion of 25% [11], a sample size of ≥1,200 patient records was needed. This study was linked with a cost-effectiveness study; we aimed to sample 2,600 medical records to meet the needs of both studies.

### Sampling

During study planning in November 2007, MOH-reported data from the end of December 2006 were used to define the clinic sample frame. By December 2006, 43,295 adults had initiated ART at 152 clinics, all of which were managed by the MOH with funding support from several donor agencies including the U.S. President's Emergency Plan for AIDS Relief (PEPFAR). Clinics that had initiated <50 adults on ART by this time were excluded from the sample frame, resulting in 58 clinics, ranging in size from 1–49 adult ART enrollees and supporting only 1,061 adult ART patients, being excluded. Ninety-four clinics, which had each enrolled between 50 and 3,530 adult ART patients, were included in the sample frame; of these clinics, 12 with >1,000 enrollees were selected with certainty, while 18 with 50–1,000 enrollees were selected using probability-proportional-to-size sampling. All 11 provinces in Mozambique were represented by the sample with at least one ART clinic selected from each province. From the 30 selected clinics, ranging in site size from 70–3,530 adult ART enrollees, we aimed to randomly sample 2,600 medical records.

### Analytic Methods

Data were analyzed using SAS 9.2 (SAS Institute Inc., Cary, NC), STATA 10 (StataCorp, 2009, Stata Statistical Software, Release 10, College Station, TX), and SUDAAN (Research Triangle Institute, 2005, SUDAAN, Release 9.0.1. Research Triangle Park, NC). Data were weighted and survey design controlled for, during analysis.

Multiple imputation with chained equations was used to impute missing baseline demographic and clinical data [24]. The ice [25]–[27] procedure in Stata was used to create 20 imputed datasets for each of two outcomes included in multivariable analysis: Attrition and treatment failure. The imputation model included the event indicator, all study variables, and the Nelson-Aalen estimate of cumulative hazard [28]. Missing data were assumed *missing at random* (MAR) and all patients had complete time-to-event data. When comparing baseline characteristics among groups, the *χ* ^2^ and *t*-test were used to compare categorical and continuous variables, respectively. Kaplan-Meier curves were used to examine retention proportions stratified by baseline variables. Cox proportional hazards regression models were used to estimate unadjusted and adjusted hazard ratios (AHRs), 95% CIs, and *p*-values. The proportional hazards assumption was assessed separately for each imputed dataset using visual methods and the Grambsch and Therneu test [29]. Estimates were combined across the imputed datasets according to Rubin's rules [24]. This was accomplished using SUDAAN and the mim procedure in Stata [30].

As a secondary analysis, mean CD4 count and weight change over time was estimated to allow comparison with published literature. SAS PROC MIXED was used to fit unweighted polynomial growth curve models with maximum likelihood (ML) estimation to the data [31]. Random effects were specified on the intercept and slope terms. Predicted means and 95% CIs from the polynomial growth curve models were plotted to graphically illustrate change over time.

## Results

### Characteristics at ART Initiation

Data from medical records of 2,596 eligible, adult ART patients were abstracted and analyzed. Table 1 illustrates analysis results for both original and imputed datasets; in the following text, weighted imputed data are reported unless otherwise stated.

**Table 1:** Demographic and clinical characteristics of adult patients at ART initiation – Mozambique, 2004–2007.

	Original Data				Following MultipleImputation (N = 2,596)	
	Un-weighted Frequency of Observations	Un-weighted Total	Weighted Median with IQR*, or, Percentage with 95% CI		Weighted Median with IQR*,or, Percentage with 95% CI	
**Median Age** † **, No., N, median (IQR)**						
Both Sexes	2,596	2,596	**34**	(28–42)	**34**	(28–42)
Female	1,576	1, 576	**32**	(27–39)	**32**	(27–39)
Male	1,020	1,020	**38**	(31–45)	**38**	(31–45)
**Female** † **, No., N, %, (95% CI)**	1,576	2,596	**62%**	(59–65%)	**62%**	(59–65%)
**Employment status, No., N, %, (95% CI)**						
Employed	992	2,268	**46%**	(40–51%)	**45%**	(40–51%)
Student	107	2,268	**4%**	(3–5%)	**4%**	(3–5%)
Unemployed	1,169	2,268	**50%**	(45–56%)	**51%**	(46–56%)
observations missing data**	328	2,596	**13%**			
**Active TB, No., N, %, (95% CI)**	267	2,564	**11%**	(9–13%)	**11%**	(9–13%)
observations missing data**	32	2,596	**1%**			
**WHO Stage, No., N, %, (95% CI)**						
I/II	619	1,617	**37%**	(32–42%)	**40%**	(34–45%)
III	739	1,617	**47%**	(43–52%)	**45%**	(40–50%)
IV	259	1,617	**16%**	(13–18%)	**15%**	(13–18%)
observations missing data**	979	2,596	**38%**			
**Weight Category, No., N, % (95% CI)**						
<45 kg	367	2,061	**17%**	(14–20%)	**18%**	(15–21%)
45–60 kg	1,224	2,061	**59%**	(56–61%)	**57%**	(54–59%)
> 60 kg	470	2,061	**24%**	(21–27%)	**25%**	(22–28%)
observations missing data**	535	2,596	**21%**			
**BMI<18.5, No., N, %, (95% CI)**	354	1,200	**28%**	(23–33%)	**28%**	(23–33%)
observations missing data**	1,396	2,596	**54%**			
**CD4^+^ T-cell count, No., N, median (IQR)**						
Both Sexes	2,254	2,596	**155**	(76–231)	**153**	(74–231)
Female	1,373	1,576	**161**	(88–244)	**159**	(87–243)
Male	881	1,020	**141**	(59–213)	**139**	(59–213)
observations missing data**	342	2,596	**13%**			
**Hemoglobin, No., N, median (IQR)**						
Both Sexes	1,899	2,596	**10.1**	(8.8–11.6)	**10.3**	(8.8–11.7)
Female	1,182	1,576	**9.8**	(8.6–11.0)	**9.9**	(8.5–11.1)
Male	717	1,020	**11.1**	(9.0–12.5)	**11.0**	(9.2–12.5)
**First Line ART Regimens** † **, No., N, %, (95% CI)**						
D4T + 3TC + NVP/EFV	2,315	2,596	**88%**	(82–94%)	**88%**	(82–94%)
AZT + 3TC + NVP/EFV	240	2,596	**11%**	(5–16%)	**11%**	(5–16%)
D4T/AZT + 3TC + ABC	17	2,596	**<1%**	(0–1%)	**<1%**	(0–1%)
Other	24	2,596	**<1%**	(0–1%)	**<1%**	(0–1%)

Abbreviations: CI, confidence interval; IQR, interquartile range; TB, tuberculosis; WHO, World Health Organization; Kgs, kilograms; BMI, body mass index; ART, antiretroviral therapy; D4T, stavudine; 3TC, lamivudine; NVP, nevirapine; EFV, efavirenz; AZT, zidovudine; ABC, abacavir; CTX, co-trimoxazole.

*Median and IQR calculated across 20 imputed datasets.

† Variables with complete data.

**Unweighted sample estimate.

At ART initiation, median age of patients was 34 years, and 62% were female of whom 16% (95% CI, 6–25%) were pregnant. About 53% (95% CI, 50–57%) of patients were married or in a civil union. Partner sero-status was documented in 13% (95% CI, 11–15%) of all records. In un-weighted analysis, 37 (11%) of 330 tested partners were sero-negative.

Active tuberculosis (TB) was frequent at ART initiation with 11% of patients receiving TB treatment. Many patients were underweight; 18% weighed <45 kilograms (kg) at ART start. Advanced disease was common with 15% of patients diagnosed as having WHO stage IV disease.

Median CD4 count at ART initiation was 153/µL; 16% (95% CI, 14–19%) had counts below 50/µL and 50% (95% CI, 47–54%) had counts ranging from 50–200/µL. Median CD4 counts at ART initiation were lower for males than females (139/µL vs. 159/µL, p<0.01).

Median hemoglobin at ART initiation was 10.3 g/dL. Severe anemia (hemoglobin <8 g/dL) was present in 13% (95% CI, 12–15%) of patients. Median hemoglobin at ART initiation was lower for women than men (9.9 vs. 11.0 g/dL, p<0.01).

Stavudine (d4T), lamivudine (3TC), and nevirapine (NVP) or efavirenz (EFV) comprised 88% of first-line regimens. Only 31% (95% CI, 26–36%) of patients were prescribed CTX at ART initiation; 3% (95% CI, 2–3%) were not prescribed CTX due to allergy.

### Mortality and Attrition

Of 2,596 patients sampled, 164 died, 564 were LTFU, and 10 stopped ART indefinitely during 4,001 patient-years of follow-up. Median follow-up duration was 1.3 years (interquartile range, 0.7–2.2 years). Of patients who died, 56% (95% CI, 49–63%) died within 90 days of starting ART. Of patients who were LTFU, 41% (95% CI, 35–48%) were lost within 90 days of starting ART.

Mortality rates were 3.4 deaths per 100 patient-years overall (95% CI, 2.8–4.2), 12.9 deaths per 100 patient-years in the first 90 days (95% CI, 9.9–17.1), and 1.8 deaths per 100 patient-years after 90 days (95% CI, 1.3–2.4).

One-year attrition was 21% (95% CI, 17–25%) with 15% LTFU (95% CI, 11–18%), 5% dead (95% CI, 4–6%), and 1% stopping ART (95% CI, 0–3%). Attrition rates were 19.8 attritions per 100 patient-years overall (95% CI, 17.9–21.9), 57.2 attritions per 100 patient-years in the first 90 days (95% CI, 49.5–66.4), and 13.2 attritions per 100 patient-years after 90 days (95% CI, 11.7–15.0).

**Figure 1:**
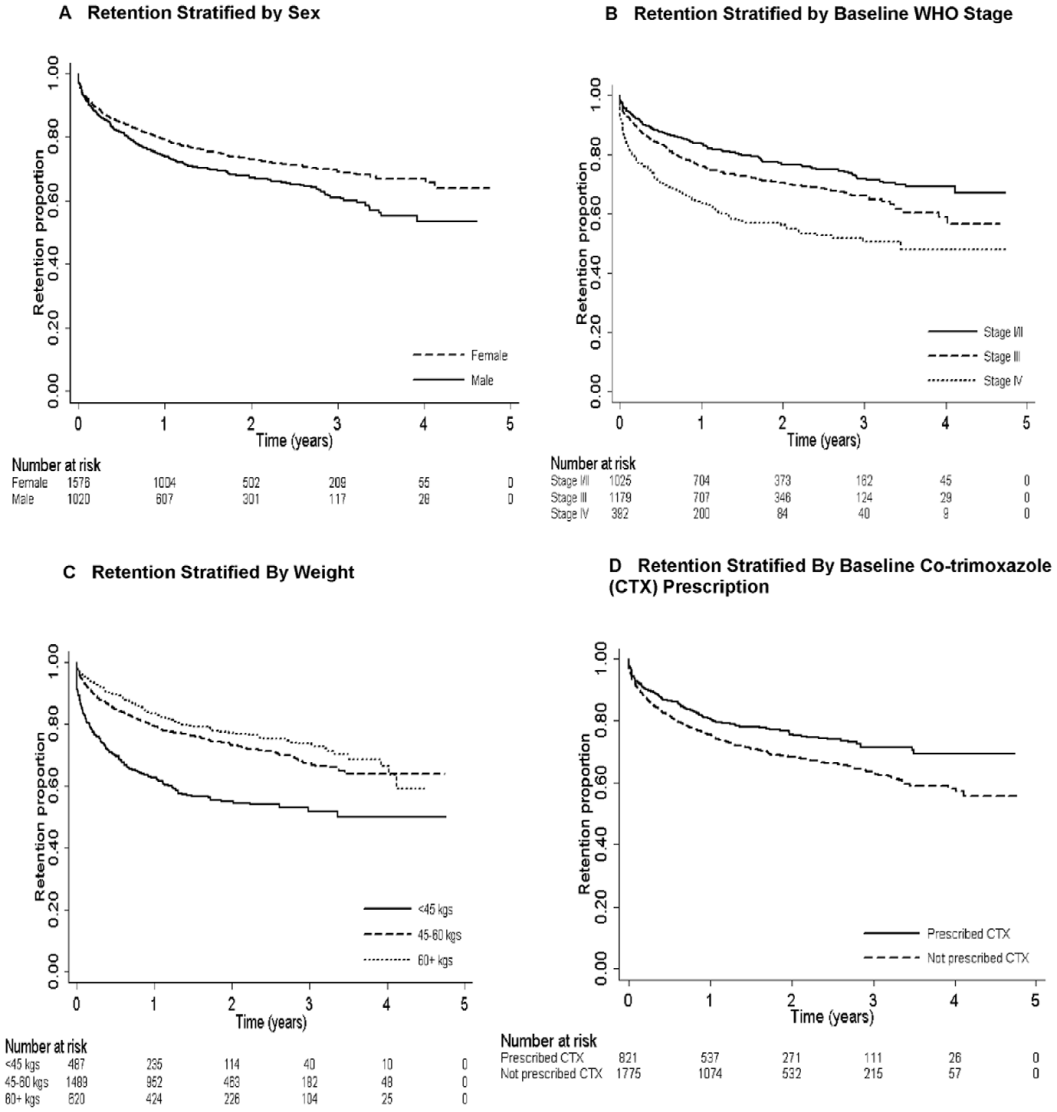
Kaplan-Meier Analysis (imputed dataset 1).

Male sex was associated with attrition (AHR 1.5; 95% CI, 1.3–1.8) (Table 2, figure 1A). Attrition risk decreased as age at ART initiation increased (AHR associated with a 10-year age increase was 0.8; 95% CI, 0.8–0.9). Patients starting ART one calendar year later had higher attrition rates (AHR 1.5; 95% CI, 1.2–1.8). Compared to patients with baseline WHO stage I/II, patients with stage IV were at higher attrition risk (AHR 1.7; 95% CI, 1.3–2.4) (Table 2, figure 1B). Patients with weight <45 kg (AHR 2.1; 95% CI, 1.6–2.9) (Table 2, figure 1C), and severe anemia (AHR 1.6; 95% CI, 1.2–2.1) were at higher attrition risk.

**Table 2:** Patient characteristics at antiretroviral therapy initiation associated with attrition and treatment failure.

	Attrition*				Immunologic Treatment Failure*			
	Original	Following Multiple Imputation (N = 2,596)			Original	Following Multiple Imputation (N = 2,596)		
		Rate/100PY	HR (95% CI)	AHR† (95% CI)		Rate/100PY	HR (95% CI)	AHR† (95% CI)
**Sex**								
Female	1,576	17.9	1.0	1.0	1,576	13.1	1.0	1.0
Male	1,020	23.0	**1.3 (1.1–1.4)**	**1.5 (1.3–1.8)**	1,020	15.5	**1.2 (1.0–1.4)**	**1.4 (1.2–1.8)**
**Age** **	2,596	–	**0.8 (0.8–0.9)**	**0.8 (0.8–0.9)**	2,596	–	**0.9 (0.8–1.0)**	**0.9 (0.8–1.0)**
**Year of ART Start** ‡	2,596	–	**1.6 (1.2–2.0)**	**1.5 (1.2–1.8)**	2,596	–	1.1 (1.0–1.2)	1.1 (0.9–1.2)
**Married**								
Married/Living together	1,211	21.2	1.0	1.0	1,211	12.4	1.0	1.0
Single/Widowed	1,152	18.3	0.9 (0.8–1.0)	0.9 (0.8–1.1)	1,152	15.7	**1.3 (1.1–1.4)**	**1.3 (1.2–1.5)**
**Employed**								
Yes	992	18.8	1.0	1.0	992	13.4	1.0	1.0
Student	107	18.8	1.1 (0.7–1.5)	1.0 (0.7–1.4)	107	15.8	1.2 (0.9–1.5)	1.0 (0.7–1.5)
No	1,169	20.8	1.1 (0.8–1.4)	1.1 (0.9–1.3)	1,169	14.4	1.1 (0.9–1.3)	1.1 (0.8–1.4)
**Active TB**								
No	2,297	18.9	1.0	1.0	2,297	14.0	1.0	1.0
Yes	267	27.9	**1.4 (1.0–2.0)**	1.0 (0.8–1.4)	267	13.9	1.0 (0.8–1.3)	1.0 (0.7–1.3)
**WHO Stage**								
Stage I/II	619	13.3	1.0	1.0	619	14.6	1.0	1.0
Stage III	739	21.7	**1.5 (1.2**–**2.0)**	1.1 (0.8–1.6)	739	13.1	0.9 (0.7–1.2)	0.9 (0.6–1.2)
Stage IV	259	35.0	**2.5 (1.9–3.2)**	**1.7 (1.3–2.4)**	259	14.7	1.0 (0.7–1.4)	0.9 (0.6–1.3)
**Weight**								
>60	470	12.7	1.0	1.0	470	11.7	1.0	
45–60	1,224	18.1	1.4 (1.1– 1.8)	1.2 (1.0–1.6)	1,224	14.3	1.2 (1.0–1.5)	1.3 (1.0–1.7)
<45	367	41.9	2.9 (2.2–3.8)	**2.1 (1.6–2.9)**	367	17.5	1.5 (1.0–2.3)	1.6 (1.0–2.6)
**CD4^+^ T-cell count (cells/µL)**								
>200	754	18.8	1.0	–	754	16.7	1.0	–
>50–≤200	1,144	18.5	1.0 (0.8–1.3)	–	1,144	10.2	**0.6 (0.5–0.8)**	–
<50	356	25.8	**1.4 (1.1–1.8)**	–	356	22.1	1.3 (1.0–1.8)	–
**Hemoglobin**								
≥8.0 g/dL	1,664	17.2	1.0	1.0	1,664	14.0	1.0	1.0
<8.0 g/dL	235	40.5	**2.2 (1.7–2.8)**	**1.6 (1.2–2.1)**	235	14.1	1.0 (0.7–1.5)	0.9 (0.6–1.5)
**Prescribed CTX**								
Yes	821	15.3	1.0	1.0	821	13.7	1.0	1.0
No	1,775	21.9	**1.4 (1.1–1.8)**	**1.4 (1.0–1.8)**	1,775	14.1	1.0 (0.9–1.2)	1.0 (0.9–1.2)
**Adherence**								
≥95%	1,263	17.0	1.0	1.0	1,263	13.4	1.0	1.0
<95%	597	28.3	**1.5 (1.0–2.3)**	1.3 (0.8–2.0)	597	15.8	1.2 (0.9–1.6)	1.2 (0.9–1.6)
**Site Size**								
>1,000 patients	2,109	15.9	1.0	1.0	2109	13.8	1.0	1.0
≤1,000 patients	487	37.3	**2.1 (1.1–3.8)**	1.5 (0.8–2.6)	487	14.6	1.0 (0.7–1.5)	1.0 (0.6–1.5)

Abbreviations: Rate/100PY, rate per 100 person-years; HR, hazards ratio; AHR, adjusted hazards ratio; CI, confidence interval; TB, tuberculosis; WHO, World Health Organization; BMI, body mass index; CTX, co-trimoxazole.

*Stratified by CD4^+^ T-cell count (cells/µL).

† All variables listed in this table were included in the multivariate Cox proportional hazards regression model.

**Hazard ratios associated with a 10-year increase in age.

‡ Date of ART initiation was entered into the model; hazard ratios represent a yearly increase rather than a daily increase.

Patients not prescribed CTX were at higher risk for attrition compared with patients who were prescribed this drug (AHR 1.4; 95% CI, 1.0–1.8) (Table 2, Figure 1D).

### Immunologic Treatment Failure and Regimen Switch

Of 2,596 patients at risk, 486 experienced immunologic treatment failure during 3,481 patient-years of follow-up. The failure rate was 14.0 failures per 100-patient years (95% CI, 13.0-16.0). The regimen-switch rate was 0.6 per 100-patient years (95% CI, 0.4-1.1).

Predictors of immunologic treatment failure included male sex (AHR 1.4; 95% CI, 1.2–1.8) and being single or widowed (AHR 1.3; 95% CI, 1.2–1.5). Risk of immunologic failure decreased as age at ART initiation increased (AHR associated with a 10-year age increase was 0.9; 95% CI, 0.8–1.0).

### CD4 Count and Weight Response

**Figure 2:**
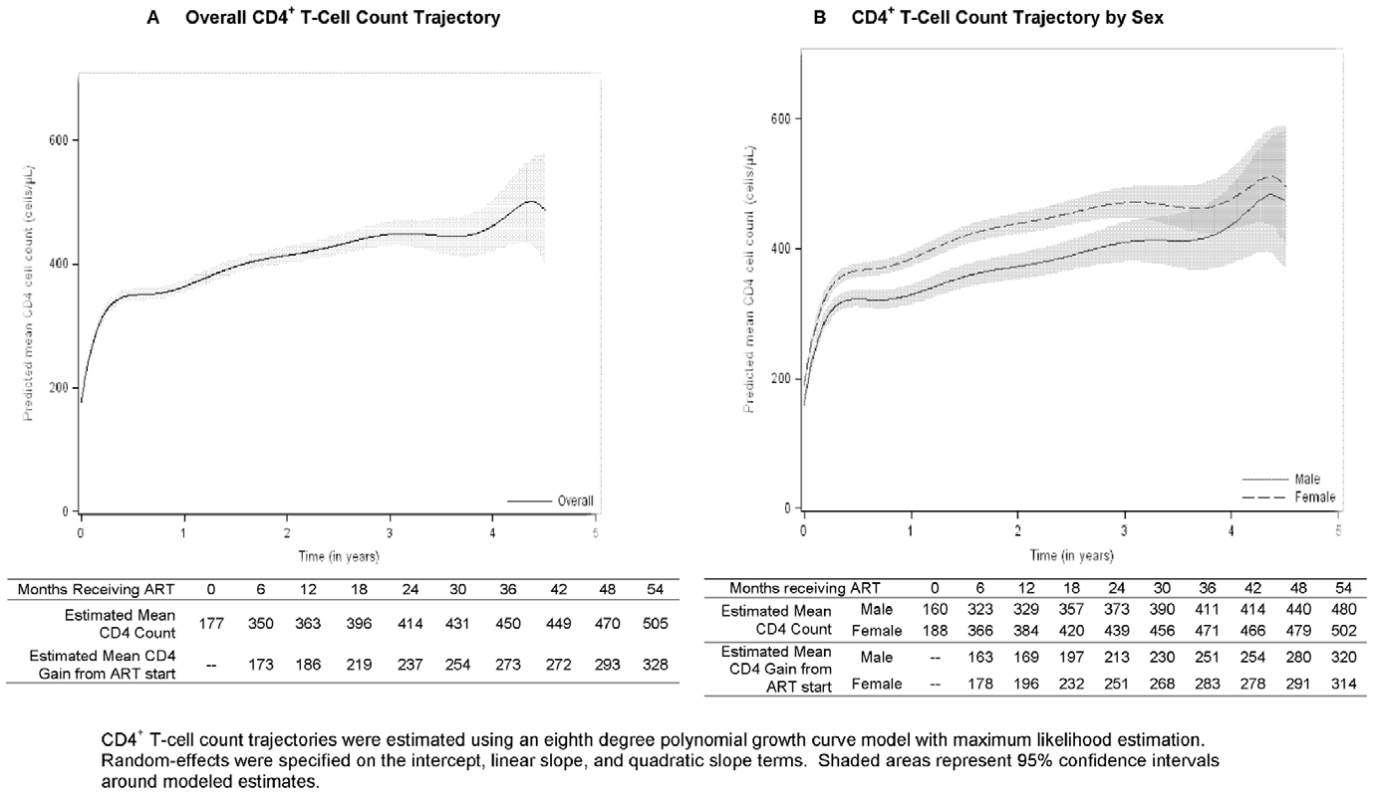
Modeled changes in CD4 count for surviving patients initiating antiretroviral therapy during 2004–2007.

**Figure 3:**
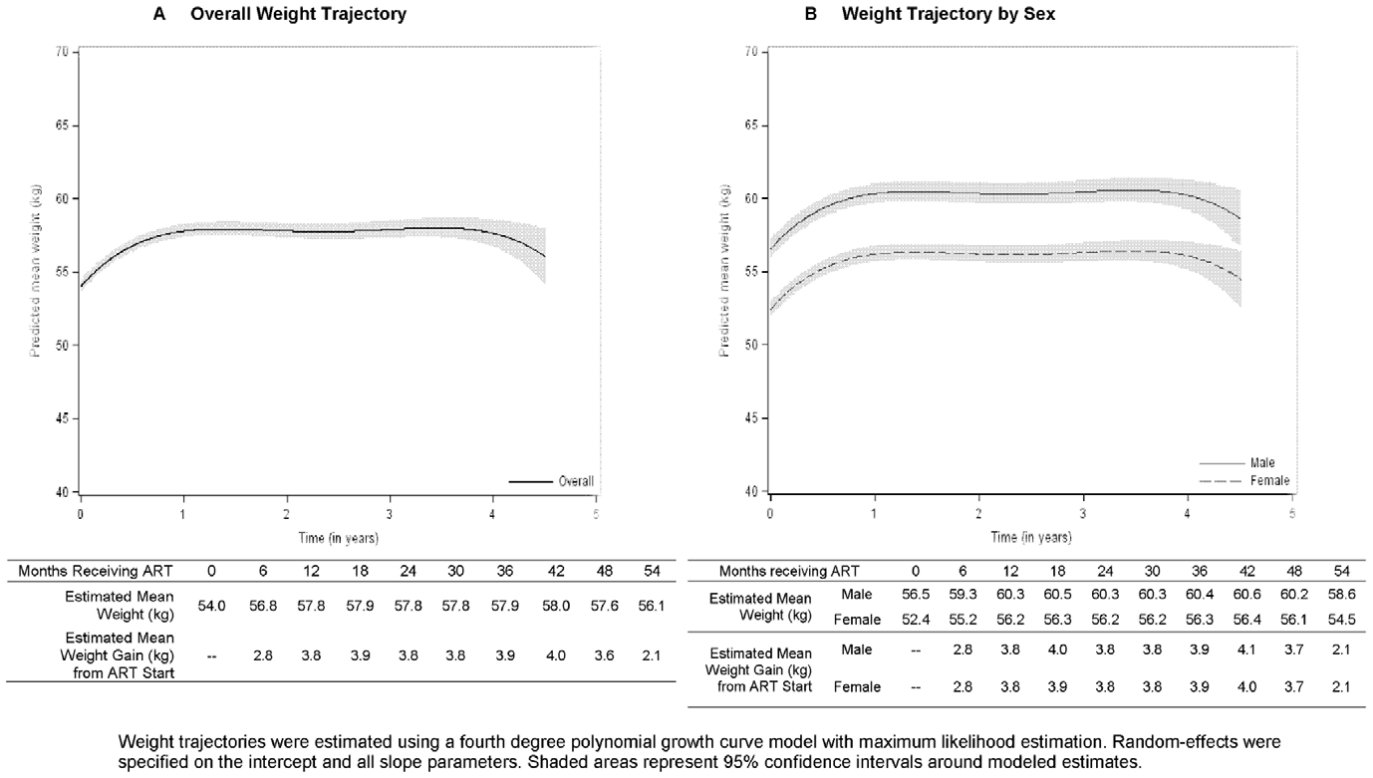
Modeled changes in weight over time for surviving patients initiating ART during 2004–2007.

Modeled mean gains in CD4 count were 173, 186, 237, 273, and 293/µL at 6, 12, 24, 36 and 48 months of follow-up, respectively (Figure 2A). Compared with males, females tended to start ART with higher CD4 counts and maintain higher counts during the first three treatment years (Figure 2B). Modeled mean weight gains were 2.8, 3.8, 3.8, 3.9 and 3.6 kilograms at 6, 12, 24, 36 and 48 months of therapy (Figure 3A) and were similar for males and females (Figure 3B).

### Adherence

About 71% (95% CI, 66–77%) of patients were ≥95% adherent to ART medicine pick-up appointments during the first six months of therapy. Proportions of female and male ART patients who were ≥95% adherent to ART were not significantly different (72% vs. 70%, p = 0.12).

## Discussion

This study adds to the limited number of nationally representative ART outcome assessments from sub-Saharan Africa [6], [12], [13], and has several important findings.

First, rates of mortality, attrition, and immunologic treatment failure for the study period are comparable with reports from other ART programs in resource-rich [9] and -constrained settings [6], [9], [11], [13], [17], [18], which is encouraging for program funders and managers. Second, regimen-switch rates were very low, a finding which deserves further research as it possibly indicates delay in switching patients from failing first-line regimens and risk of poor longer term outcomes [20]. Third, as has been reported from other ART programs, male sex, advanced HIV disease, and malnutrition predicted poor outcomes, suggesting that male-specific interventions, starting ART at earlier disease stages, and evidence-based interventions for malnourished patients, could improve future program outcomes. Fourth, CTX prescription at ART initiation, which was documented for about one third of patients, was associated with lower attrition risk, highlighting the need for CTX scale-up among ART patients [32]–[35]. Fifth, attrition rates appeared to increase as the ART program expanded, a concerning trend that requires further research to better understand underlying causes. Finally, documenting partner sero-status was uncommon, possibly indicating missed prevention opportunities among sero-discordant couples [36].

### Treatment Outcomes

Meta-analyses of attrition in adult ART programs in sub-Saharan Africa have reported mean one-year attrition proportions of 25% and 20% for the review periods of 2000-2007 [11] and 2007–2009 [18], respectively. In these studies, LTFU accounted for between 56% [11] and 59% [18] of attrition. Similarly, in our study, one-year attrition was 21% with LTFU accounting for most (71%) of patient loss from the program. This relatively high contribution of LTFU to attrition likely reflects the lack of active patient tracing systems in most Mozambican ART facilities [9], [37] due to their cost [37].

Since recent reports show that patient death may account for 29–59% of patients who are LTFU [38]–[42], we should consider our reported mortality and attrition rates as best- and worst-case estimates of true mortality. Our best-case estimate of mortality (3.4 deaths per 100 person-years) is similar to reports from adult ART programs with passive follow-up systems (2.7 deaths per 100 patient-years [9]) but predictably lower than that reported for programs with active follow-up systems (5.5 deaths per 100 patient-years [9]). Our worst case estimate for true mortality (19.8 deaths per 100 person-years) is comparable with the rate of 16.1 deaths per 100 patient-years reported for a large Zambian cohort [6].

Similar to other programs [4]–[6], [8]–[10], [13], [17], [43]–[50], large proportions of documented mortality (56%) and LTFU (41%) occurred within 90 days of starting ART, highlighting the importance of this time period. Evidence based interventions which reduce early mortality are urgently needed [51]. To limit early LTFU from the program, the MOH is considering a pilot program to rapidly trace all patients LTFU in the 90 days following ART initiation.

Post 90-day mortality and attrition rates of 1.8 and 13.2 events per 100 patient-years, respectively, are comparable with post 90-day mortality rates reported from both resource-constrained [6], [9], [17] and –rich [9] settings (5.0–6.0 deaths per 100 patient-years). Similarly, CD4 count and weight gains [6], [9], [13], [17], and rates of immunologic treatment failure [6], [17], [52], are similar to those reported from other ART programs.

### Rates of Regimen-Switch

Compared with the average regimen-switch rate for resource-constrained programs (2.4 switches per 100 person-years, 95% CI, 2.2–2.6 [20]), our switch rate (0.6 regimen changes per 100 person-years) was low. Limited access to routine viral load testing has likely contributed to low regimen-switch rates [20]. However, even compared with regimen-switch rates among programs lacking routine viral load testing (2.0 per 100 person-years, 95% CI 1.8–2.3 [20]), our switch rates are low. Limited access to second-line therapy, or training in its use, may have contributed to low switch rates, especially at more peripheral health facilities where ART is commonly managed by non-physician clinicians [20], [53]. Because low regimen-switch rates may indicate delay in switching patients from failing first-line regimens, which could lead to poor long-term outcomes [20], [54] further research to better understand the reasons for low regimen-switch rates is planned.

### Advanced HIV Disease

Similar to other reports, many patients initiated ART with WHO stage IV disease, which was a strong predictor of attrition [5], [6], [9], [44], [47], [49], [55]–[57]. This suggests that earlier diagnosis and ART is needed to improve program outcomes [51]. Mass HIV testing campaigns, earlier entry into HIV care, and ensuring pre-ART care retention, will likely be needed to reduce absolute numbers of late starters [51]. In addition, the MOH raised the CD4 count threshold for ART initiation among adults with WHO stage I/II disease from 200 to 250/µL in 2008, and from 250 to 350/µL in 2010 [58], which should reduce the proportion of patients initiating ART with end-stage disease.

### Male Sex

Similar to other reports [5], [6], [8], [10], [55], male sex was associated with poorer outcomes. In our study, males tended to initiate ART at lower CD4 counts than females, suggesting late presentation contributed to poorer outcomes. However, even in multivariate analysis, male sex was associated with poorer outcomes. Reasons for this are unknown, but could relate to differences in health-seeking or adherence behavior [59].

### Malnutrition

As in other studies, our marker for malnutrition at ART initiation (weight <45 kg [60]) was independently associated with poor outcomes. In Mozambique, the relative importance of underlying causes of malnutrition among HIV-infected persons is not well understood and deserves further research [61]. Food supplementation and empiric TB treatment for malnourished patients are interventions which require evaluation [62].

### Failure to Prescribe CTX

Although CTX prescription is recommended for all ART patients in Mozambique throughout therapy, few (31%) received this drug at ART initiation during 2004–2007. Reasons for this are unknown but may include CTX supply shortfalls and clinician concerns about CTX drug resistance development [32], [33], [63].

Although several studies have demonstrated an additional benefit of CTX for ART patients [32]–[35], reasons for this are not fully understood [32]. In Mozambique, where malaria is an important cause of mortality among ART patients [64], the prophylactic action of CTX against this disease [32], may partly explain its association with improved treatment outcomes.

Additional in-service training for ART-service providers is planned to rapidly scale-up CTX prescription. Further, CTX procurement and distribution procedures are being reviewed to ensure that all ART delivery sites have adequate CTX supplies.

### Temporal Trends in Attrition

As the ART program expanded during 2004 through 2007, attrition risk for newly enrolled adult ART patients increased, a temporal trend which has been observed in other programs in resource-constrained settings [65], [66]. Yearly increases in observed LTFU due to undocumented transfer of patients to more peripheral facilities during the decentralization process, may explain the increases in observed attrition rates [67], [68]; however, further research to establish the reasons for increasing rates of attrition is urgently needed [65].

### Partner HIV Counseling and Testing

The low proportion of records with documented partner sero-status is concerning because partner HIV counseling and testing (HIV-CT) can facilitate entry into HIV care for infected individuals or appropriate prevention strategies for uninfected partners [36], [69]–[76]. Poor completion of the partner sero-status field in standard MOH medical records may be due to several barriers including hesitancy of patients to disclose HIV status, partner refusal to be tested, or loss to follow-up of the patient before the field can be filled. Further research to identify and overcome these barriers is needed.

### Limitations

Limitations primarily relate to the fact that these analyses were based on routinely collected data, which were incomplete for certain baseline and follow-up clinical characteristics.

### Summary

Treatment outcomes were comparable with reports from other ART programs; however, the low ART regimen-switch rates and the increasing attrition rates during program expansion are concerning findings which deserve further research. Immediate public health responses that may improve treatment outcomes include initiation of ART at earlier disease stages, especially among males, scale-up of CTX, and evidence-based interventions for malnourished patients; in addition, scale-up of partner HIV-CT could significantly contribute to national HIV prevention efforts.
